# Serum-derived factors of breast cancer patients with brain metastases alter permeability of a human blood–brain barrier model

**DOI:** 10.1186/s12987-020-00192-6

**Published:** 2020-04-22

**Authors:** Carolin J. Curtaz, Constanze Schmitt, Saskia-Laureen Herbert, Jonas Feldheim, Nicolas Schlegel, Fabien Gosselet, Carsten Hagemann, Norbert Roewer, Patrick Meybohm, Achim Wöckel, Malgorzata Burek

**Affiliations:** 1 Department of Gynecology and Obstetrics, University of Würzburg, Würzburg, Germany; 2 Department of Anaesthesia and Critical Care, University of Würzburg, Oberdürrbacher Straße 6, 97080 Würzburg, Germany; 3 Department of Neurosurgery, Tumour Biology Laboratory, University of Würzburg, Würzburg, Germany; 4 Department of Surgery I, University of Würzburg, Würzburg, Germany; 5 Blood–Brain Barrier Laboratory, Université d’Artois, UR 2465 Lens, France

**Keywords:** Metastatic breast cancer, Blood–brain barrier, In vitro models, CX3CL1, CXCL13

## Abstract

**Background:**

The most threatening metastases in breast cancer are brain metastases, which correlate with a very poor overall survival, but also a limited quality of life. A key event for the metastatic progression of breast cancer into the brain is the migration of cancer cells across the blood–brain barrier (BBB).

**Methods:**

We adapted and validated the CD34^+^ cells-derived human in vitro BBB model (brain-like endothelial cells, BLECs) to analyse the effects of patient serum on BBB properties. We collected serum samples from healthy donors, breast cancer patients with primary cancer, and breast cancer patients with, bone, visceral or cerebral metastases. We analysed cytokine levels in these sera utilizing immunoassays and correlated them with clinical data. We used paracellular permeability measurements, immunofluorescence staining, Western blot and mRNA analysis to examine the effects of patient sera on the properties of BBB in vitro.

**Results:**

The BLECs cultured together with brain pericytes in transwells developed a tight monolayer with a correct localization of claudin-5 at the tight junctions (TJ). Several BBB marker proteins such as the TJ proteins claudin-5 and occludin, the glucose transporter GLUT-1 or the efflux pumps PG-P and BCRP were upregulated in these cultures. This was accompanied by a reduced paracellular permeability for fluorescein (400 Da). We then used this model for the treatment with the patient sera. Only the sera of breast cancer patients with cerebral metastases had significantly increased levels of the cytokines fractalkine (CX3CL1) and BCA-1 (CXCL13). The increased levels of fractalkine were associated with the estrogen/progesterone receptor status of the tumour. The treatment of BLECs with these sera selectively increased the expression of CXCL13 and TJ protein occludin. In addition, the permeability of fluorescein was increased after serum treatment.

**Conclusion:**

We demonstrate that the CD34^+^ cell-derived human in vitro BBB model can be used as a tool to study the molecular mechanisms underlying cerebrovascular pathologies. We showed that serum from patients with cerebral metastases may affect the integrity of the BBB in vitro, associated with elevated concentrations of specific cytokines such as CX3CL1 and CXCL13.

## Introduction

The blood–brain barrier (BBB) is a natural barrier that specializes in protecting the brain from harmful substances, including anti-tumour drugs. In vitro models can help to better understand the mechanisms and changes at the BBB in cancer patients and can provide information for future therapies. In vitro BBB models have been used over the past five decades to investigate molecular mechanisms underlying physiological and pathological processes at the BBB and central nervous system (CNS) and to perform drug discovery studies. Central to these models are the brain microvascular endothelial cells, which form a tight barrier through cell–cell contacts, tight, adherens and gap junctions. Specific transporter and receptors are responsible for supplying the brain with energy and clearing it of toxic substances. Rat, mouse, pig and bovine in vitro models are commonly used to study BBB characteristics [1, 2]. Human primary brain microvascular endothelial cells rapidly lose their properties when removed from their natural environment [3] and show high permeability when immortalized [1]. The development of stem cell-based technologies opened up a new opportunity to generate human in vitro BBB models. Induced pluripotent stem cells (iPSCs)-derived brain-like endothelial cells (BLECs) can be obtained by specific differentiation protocols [4–11]. Another published method is the use of umbilical cord blood-derived hematopoietic stem cells for differentiation into endothelial cells, followed by the induction of BBB properties by co-culture with brain pericytes [12–14]. These advances in in vitro BBB modelling contribute to advances in the understanding of CNS disorders, such as brain metastases of breast cancer [15–17].

Breast cancer is the world’s most common malignant tumour in women and causes the highest tumour-related death among them in western industrialised countries. Fortunately, nowadays, primary breast cancer is a well treatable disease with high overall survival. In contrast, tumour metastases such as visceral, bone and cerebral metastases at later stages of this cancer play an important role for overall survival and mortality. However, appearance of metastases also means that a level of systemic tumour disease was reached and therefore describes a palliative situation. Overall, about 10–15% of the breast cancer patients develop cerebral metastases, one of the most severe metastases types, which is associated with a very poor prognosis with only 20% one-year survival [18]. Particularly, histological cancers of the triple-negative (estrogen receptor, progesterone receptor and epidermal growth factor receptor 2 negative) and epidermal growth factor receptor 2 (HER2/neu) subtypes have a high potential for cerebral metastases [19, 20]. The mechanism of metastatic progression of breast cancer into the brain and the migration of cancer cells via the BBB remains not well understood in detail.

More than 40 chemokines, small proteins of 8–14 kDa, were identified in humans. They are grouped into four classes based on the position of their N-terminal cysteine residues: CC, CXC, XC and CX3C. They play a role in numerous biological processes like immune system homeostasis, cell proliferation and differentiation. Recent evidence indicates that chemokines also play a role in cancer progression and metastatic dissemination of solid tumours [21].

In the present study, we used a CD34^+^ cells-derived in vitro BBB model in co-culture with brain pericytes to study the differential effects of breast cancer patient sera on BBB properties. We analysed chemokine levels in the sera of breast cancer patients with primary cancer, bone, visceral or cerebral metastases relative to a healthy control group. We identified two chemokines that were selectively elevated in the group with cerebral metastases and these sera displayed impairing effects towards the BBB integrity in vitro.

## Materials and methods

### Patient samples

All human samples were collected from donors after signing an informed consent form in accordance with German legislation rules. Hereby, we followed and strictly adhered to the Ethical Guidelines of the University of Würzburg (reference number: 137/18-me), which are in accordance to the Helsinki Declaration of 1975 and its revision of 1983. We collected blood samples from 103 patients with breast cancer and a healthy control group of 15 women (Table 1). These samples where pseudonymised and classified into five distinct groups: cancer-free individuals (healthy control, C, n = 15), breast cancer patients with primary cancer (PC, n = 26), breast cancer patients with visceral (VM, n = 30), bone (BM, n = 20) and cerebral metastases (CM, n = 12). The blood specimen were centrifuged at 2000 g, the sera collected and stored at − 80 °C until use.

**Table 1:** Summary of clinical data

	C	PC	VM	BM	CM
Patient characteristics					
Total number	15	26	30	20	12
Median age	66	57.6	60	66.3	62.1
Deceased					5
Pre-/postmenopausal	1/14	10/16	6/24	2/18	2/10
Tumour characteristics					
ER/PR positive		15	17	16	1
HER2/neu positive		8	11	3	11
Triple negative		2	2		
Grading					
Well differentiated (G1)		2	4	0	0
Moderately differentiated (G2)		18	11	16	6
Poorly differentiated (G3)		6	15	4	6
% of Ki67 positive cells (median)		25%	40%	15%	30%
Other					1

*BM* bone metastases, *C* control group of healthy donors, *CM* cerebral metastases, *ER* estrogen receptor, *Her2/neu* human epidermal growth factor 2, *PC* primary cancer, *PR* progesterone receptor, *VM* visceral metastases

### Brain pericytes

The brain pericytes have been isolated and purified as previously published [22, 23]. Briefly, brain capillaries were isolated from bovine brain as previously described [24]. Then, these microvessels were mechanically dissociated and immediately dispatched into 12 Matrigel-coated dishes containing DMEM supplemented with 20% FCS, 2 mM l-glutamine, 50 g/mL gentamicin, and 1 ng/mL basic fibroblast growth factor (bFGF). On the following day, 60 mm Petri dishes were carefully screened for large vessels and each of them was scraped and discarded. Pericytes that migrated out of the capillaries rapidly divided and invaded the whole surface of the dish. Confluent cultures were dissociated with 0.05% trypsin/0.02% EDTA saline buffer (Biochrom AG, Berlin, Germany), and cells were frozen in liquid nitrogen. At last, brain pericytes were thawed and immortalized with the SV40 antigen.

### CD34^+^ cells-derived human in vitro BBB model

The written informed consent was obtained from the infants’ parents prior to collection of the infants’ umbilical cord blood. CD34^+^ hematopoietic stem cells-derived human endothelial cells were isolated and purified as described previously and adapted in our laboratory using already published procedures. The cells were grown in 6- or 12-well transwells in monoculture or co-culture with brain pericytes for 6 days to induce BBB-like properties and are termed brain-like endothelial cells (BLECs) [12, 14, 25]. Shortly, 80 × 10^3^ or 325 × 10^3^ BLECs were cultured on Matrigel coated 12- or 6-well transwell inserts respectively (pore size 0.4 µm, Corning) for two days in Microvascular Endothelial Cell Growth Medium (ECM) (PLEOBiotech) supplemented with 5% fetal calf serum (FCS). Brain pericytes [12] were cultured on gelatine-coated plates in DMEM (Sigma) supplemented with 20% FCS, 2 mM l-glutamine and 50 µg/ml gentamycin. For co-cultures, 50 × 10^3^ brain pericytes were seeded into 12-well plates and grown in ECM along with the BLECs on transwell inserts for 5 days to induce BBB-characteristics of the latter. Endothelial cells grown alone or in co-culture with brain pericytes were used to determine paracellular permeability and to isolate RNA and protein. For the incubation experiments, BLECs were treated with ECM supplemented with 2% patient sera for 24 h before transcriptional, immunofluorescent and permeability studies.

### Immunofluorescence

CD34^+^-derived endothelial cells were grown on their own or in co-culture with pericytes as described above, with or without 2% patient serum in the medium for 24 h. Separate treatments were done with individual sera. Cells were stained on transwell inserts with anti-claudin-5 antibody, conjugated to Alexa Fluor 488 (Thermo Fisher Scientific) as described previously [26].

### Real-time PCR

Real-time PCR was performed as previously described [26–28]. Briefly, RNA was isolated using the RNA isolation kit NucleoSpin^®^ RNA (Machery-Nagel) according to manufacturer’s instruction. Total RNA (500 ng) was reverse transcribed using the High Capacity cDNA Reverse Transcription Kit (Thermo Fisher Scientific). The commercially available TaqMan probes Hs00170162_m1 (OCLN), Hs00533949_s1 (CLDN5), Hs00184500_m1 (ABCB1), Hs00988717_m1 (ABCC4), Hs01053790_m1 (ABCG2), Hs00892681_m1 (SLC2A1), Hs00355476_m1 (CCL20), Hs03676656_m1 (CXCL12), Hs00234140_m1 (CCL2), Hs00757930_m1 (CXCL13), Hs00173527_m1 (CXCR5), Hs00171086_m1 (CX3CL1) and Hs00365842_m1 (CX3CR1) were used with the TaqMan^®^ Fast Advanced Master Mix in the StepOne-Plus Real-Time PCR System (Thermo Fisher Scientific). Calnexin (CANX) (Hs01558409_m1) was used for normalization. Relative expression was calculated by the comparative Ct method.

### Western blot analysis

Western blot was performed as recently described [29, 30]. Twenty micrograms of protein were subjected to SDS‐PAGE followed by transfer to a PVDF membrane (Bio-Rad Laboratories) and blocked with 5% (w/v) non-fat milk in phosphate buffered saline (PBS, pH 7.4). The membranes were incubated with the respective primary antibodies diluted in PBS containing 1% Bovine Serum Albumin (BSA, pH 7, Sigma) at 4 °C overnight. Following antibodies were used: anti-BCRP (1:100, Abcam, #Ab-24114), CLDN5 (1:500, Thermo Fisher Scientific #35-2500), GLUT-1 (1:2000, Millipore, #07-1401), MCT1 (1:200, Santa Cruz Biotechnology, #sc-14917), MRP4 (1:500, ENZO, #ALX-801-039-C100), LRP1 (1:1000, Abcam, #Ab92544), P-Glycoprotein (P-GP, 1:200, Santa Cruz Biotechnology, #sc55510), RAGE (1:200, Santa Cruz Biotechnology, #sc-8230), Transferrin Receptor (TFR, 1:500, Thermo Fisher Scientific, #13-6800). After incubation with respective secondary antibodies, images were taken using Enhanced Chemiluminescence solution and FluorChem FC2 Multi-Imager II (Alpha Innotech). The intensity of protein bands was measured with ImageJ software.

### Endothelial permeability measurements

Coated transwell inserts and inserts containing confluent BLECs were placed into a plate with 1.5 ml HEPES-buffered Ringer’s solution (pH 7.4). 500 µl of the same solution containing 1 mM fluorescein (Sigma) was added to the upper compartment. During the 1-hour assay, the inserts were placed into a new well with fresh buffer solution every 20 min and aliquots from the receiver compartment were collected. Aliquots from the donor solution were taken at the beginning and at the end of the experiment. All samples were measured at 490/516 nm wavelengths using a microplate reader Tecan Microplate Reader (Thermo Fisher Scientific). For each treatment condition, at least three inserts with and without cells were tested.

### Cell viability measurement

BLECs were grown to confluence in a 96-well plate and were treated with recombinant human BCA-1 (PeproTech, #300-47) or/and fractalkine (PeproTech, #300-31) in concentrations of 0.5, 5, 50 and 500 ng/ml for 24 h. After the incubation, 100 µl MTT solution (1 mg/ml, Sigma) prepared in DMEM without phenol red was added and incubated at 37 °C for 4 h. Untreated cells were used as a control. The MTT solution was removed and the formazan crystals were solubilized with 100 µl of isopropanol followed by absorbance measurement at 560 nm and 690 nm using Tecan Microplate Reader (Thermo Fisher Scientific).

### Cytokine immunoassay

Concentrations of human CCL2, CCL5, CCL20, CXCL12, CXCL13 and CX3CL1 were measured in duplicate in individual patient sera using a multi-analyte immunoassay and Luminex^®^ bead technology with reagent kits (Merck Millipore) according to manufacturer’s instructions.

### Statistical analysis

GraphPad Prism 7 (GraphPad Software) was used for statistical analyses. Data are expressed as mean ± standard deviation (SD) unless otherwise stated. Differences among groups were analysed either using the ANOVA with Tukey’s multiple comparison test or a non-parametric Kruskal–Wallis test with Dunn’s multiple comparison post hoc test. Mann–Whitney U test was used to compare two groups. Statistical significance was assumed for p < 0.05. The Spearman-Rho correlation coefficients were calculated for any two measurements of chemokines and of chemokines and tumour properties within the patient population (n = 103). Correlation analysis was performed using the IBM SPSS Statistics 23 Software (IBM Corporation).

## Results

### Validation of human in vitro BBB model

**Fig. 1:**
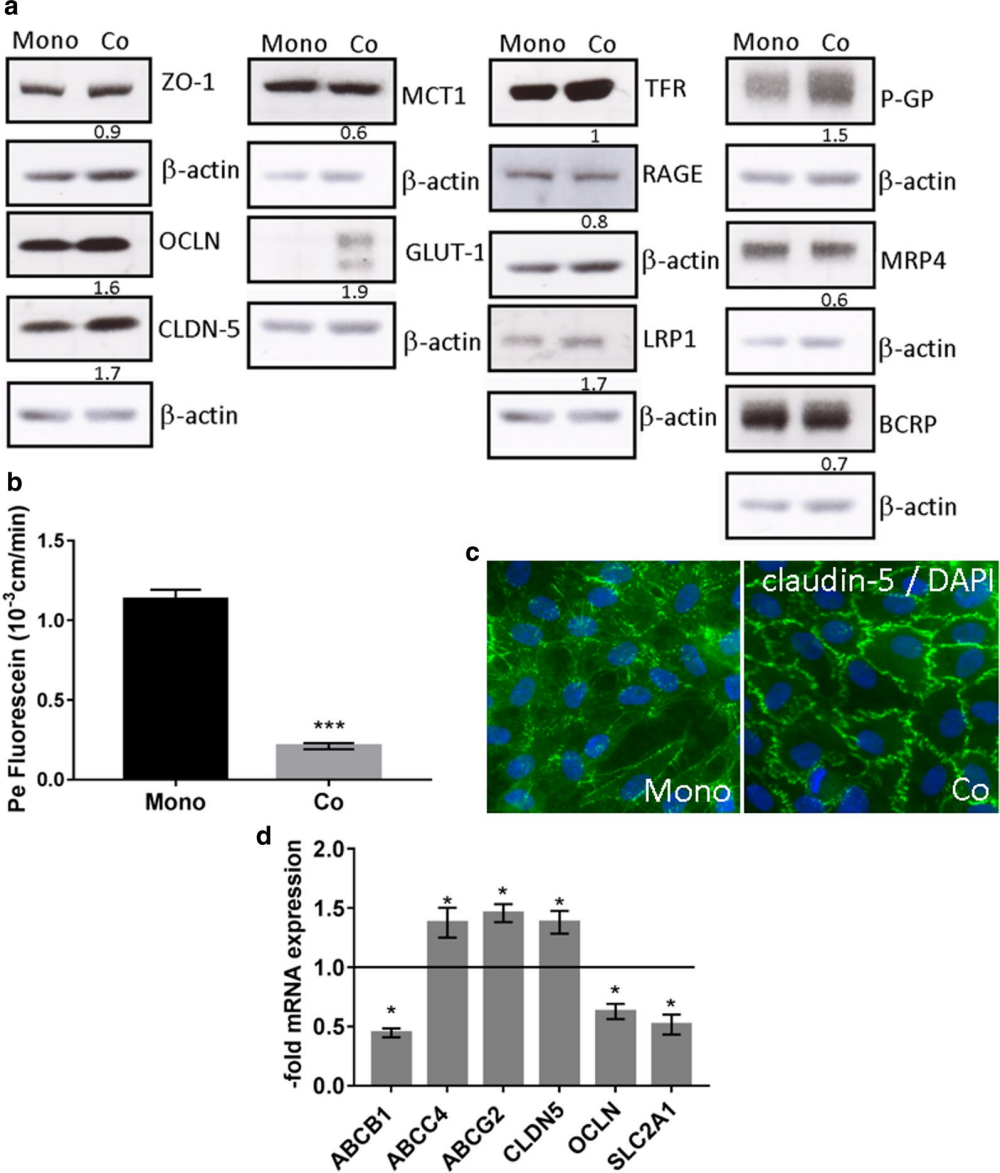
BLECs in co-culture with pericytes develop a tight barrier. CD34^+^-derived endothelial cells were cultured as monoculture or co-culture with brain pericytes for 6 days in a transwell system. **a** Expression of selected BBB marker proteins was induced in co-culture (Co) in comparison to monoculture (Mono) as shown by Western blot. Numbers under the representative bands indicate protein levels normalised to β-actin and to monoculture control. **b** Paracellular permeability for fluorescein of CD34^+^-derived endothelial cells monolayers either cultured alone or together with pericytes. Data are shown as mean ± SD (n = 3), ***p < 0.001. **c** Expression of claudin-5 in CD34^+^-derived endothelial cells cultured alone or in co-culture with brain pericytes shown by immunofluorescence. Magnification ×400, green: claudin-5, blue: DAPI nuclear staining. **d** Messenger RNA expression of transporters and tight junction proteins in BLECs was quantified by qPCR. Target gene expression was normalized to endogenous control and shown as fold over control, which was arbitrarily set as 1 (control level marked in graph). Data are shown as mean ± SD of three experiments. *p < 0.05 statistically significant versus control. *BCRP* breast cancer resistance protein (ABCG2), *CLDN5* claudin-5, *GLUT-1* glucose transporter type 1 (SLCA1), *LRP1* LDL receptor related protein 1, *MCT1* monocarboxylate transporter 1 (SLC16A1), *MRP4* multidrug resistance-associated protein 4 (ABCC4), *OCLN* occludin, *P-GP* P-glycoprotein 1 (ABCB1), *RAGE* receptor for advanced glycation end-products (AGER), *TFR* transferrin receptor (TFRC), *ZO1* zonula occludens 1 (TJP1)

After 6 days co-culture with brain pericytes expression of TJ proteins, occludin and claudin-5 was induced in BLECs (Fig. 1a). The solute carrier transporter GLUT-1 was also induced. Among the cellular receptors and efflux pumps, only the LRP-1 and P-GP showed induction. Other analysed proteins were slightly downregulated (Fig. 1a). Measurement of paracellular permeability of fluorescein (400 Da) yielded low permeability values (0.21 ± 0.02 × 10^−3^ cm/min), which were more than 80% lower than in CD34^+^-derived endothelial cells monoculture (1.13 ± 0.06 × 10^−3^ cm/min), (Fig. 1b). In addition, the endothelial cells were stained with anti-claudin-5 antibody (Fig. 1c). Only BLECs in co-culture with pericytes formed a monolayer with the correct localization of claudin-5 at the TJs. Next, mRNA expression of BBB markers was evaluated. All analysed transcripts were significantly altered in BLECs co-cultured with pericytes compared to CD34^+^-derived endothelial cells monoculture (Fig. 1d). While ABCB1, SLC2A1 and occludin mRNAs were downregulated, ABCC4, ABCG2 and claudin-5 mRNAs were significantly upregulated. There is a discrepancy between protein and mRNA levels for GLUT-1, P-gp and OCLN. Protein levels are induced due to co-culture, while mRNA levels are reduced. The reason for this could be posttranscriptional regulation that can interfere with translation, e.g. regulation by microRNA [31]. These results indicate that co-culture with brain pericytes is indispensable for the induction of BBB-properties in CD34^+^-derived endothelial cells.

### Quantification of chemokines in patient serum

**Fig. 2:**
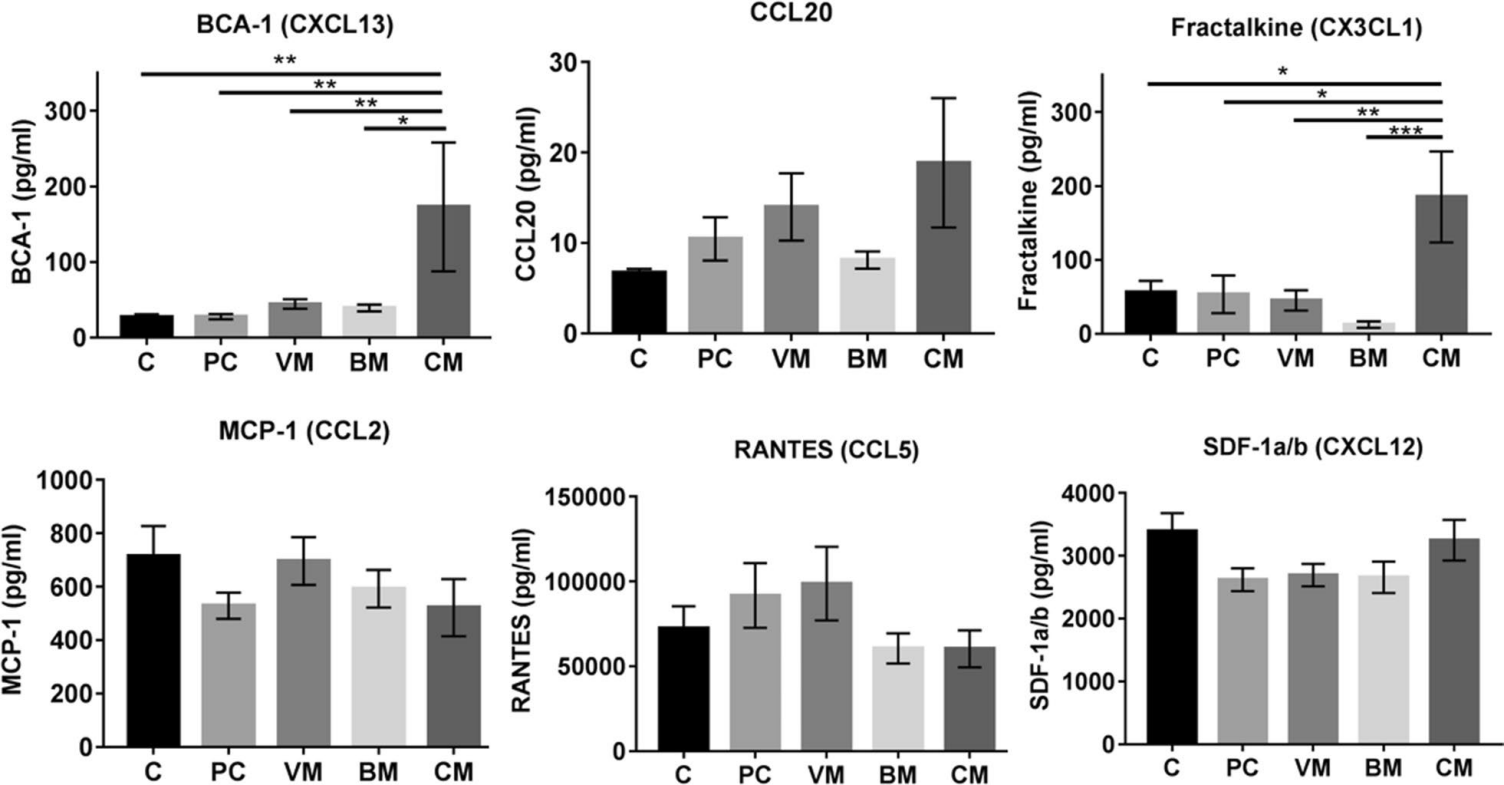
Serum levels of cytokines and chemokines measured in serum from healthy donors and breast cancer patients. Cytokines and chemokines were measured in the sera by multi-analyte immunoassay in following groups: persons without cancer (C), breast cancer patients with primary cancer (PC), breast cancer patients with visceral (VM), bone (BM) and cerebral metastases (CM). Data are presented as mean ± SEM. *p < 0.05, **p < 0.01, ***p < 0.001

**Fig. 3:**
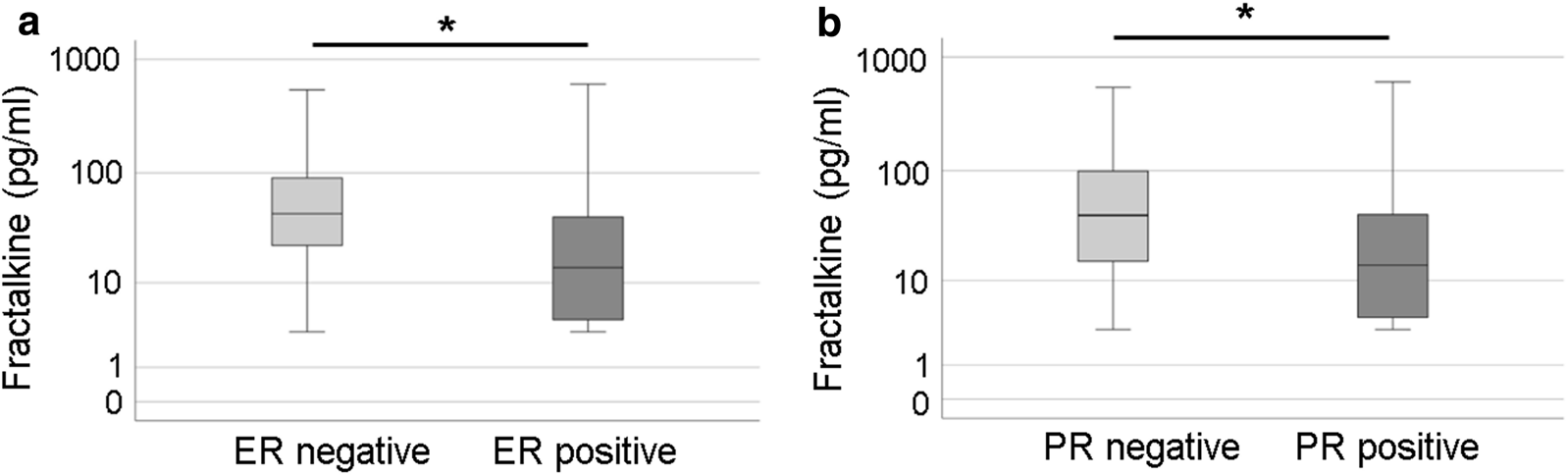
Box plots of fractalkine serum concentrations in patients with different estrogen and progesterone receptor status of the tumour. The values of fractalkine concentrations (pg/ml) are shown on a logarithmic scale. *ER* estrogen receptor, *PR* progesterone receptor, *p < 0.05

We compared the concentration of selected chemokines in the sera of healthy controls (C), breast cancer patients with primary cancer (PC), breast cancer patients with visceral (VM), bone (BM) and cerebral metastases (CM) using an immunoassay (Fig. 2). Mean serum concentrations of BCA-1 (CXCL13) and fractalkine (CX3CL1) were significantly higher in the sera of breast cancer patients with cerebral metastases compared to all other patient populations or controls. CCL20 showed a similar tendency. No differences between the groups were found for the mean serum concentrations of MCP-1 (CCL2), RANTES (CCL5) and SDF-1a/b (CXCL12) (Fig. 2). In the patient population a statistically significant correlations between MCP-1 and RANTES (r = 0.238, p = 0.04), BCA-1 and CCL20 (r = 0.362, p < 0.01), and histological grading and Ki67 staining (r = 0.456, p < 0.01) were seen (Table 2). Patients with ER and PR negative tumours had statistically significant higher fractalkine concentrations in serum (Fig. 3). A statistically significant negative correlation between BCA-1 concentrations and the histological grading (r = − 0.808, p = 0.003) was seen in patients with cerebral metastases (n = 12).

**Table 2:** Correlation analysis of chemokine concentrations and tumour characteristics

Correlates with:		MCP-1 (pg/ml)	RANTES (pg/ml)	SDF-1a/b (pg/ml)	BCA-1 (pg/ml)	CCL20 (pg/ml)	Grading	Ki67 staining
Fractalkine (pg/ml)	r	− 0.03	0.09	0.18	0.19	0.04	0.13	0.18
	p	0.83	0.45	0.12	0.11	0.73	0.33	0.20
MCP-1 (pg/ml)	r		0.238*	0.13	− 0.12	0.00	− 0.04	0.02
	p		0.04	0.26	0.29	0.99	0.79	0.87
RANTES (pg/ml)	r			0.20	0.04	0.05	− 0.09	0.19
	p			0.05	0.68	0.60	0.44	0.11
SDF-1a/b (pg/ml)	r				0.02	− 0.12	− 0.09	0.17
	p				0.83	0.24	0.41	0.15
BCA-1 (pg/ml)	r					0.362**	− 0.04	0.08
	p					< 0.01	0.72	0.51
CCL20 (pg/ml)	r						0.00	0.15
	p						0.98	0.24
Grading	r							0.456**
	p							< 0.01

*r* correlation coefficient (the positive “r” means positive correlation, while the negative “r” means negative correlation; the closer r value to 1 or − 1, the greater the correlation)

p: p value indicating statistical significance

*p < 0.05; **p < 0.01

### Patient serum and recombinant chemokines lead to reduced barrier properties of BLECs

**Fig. 4:**
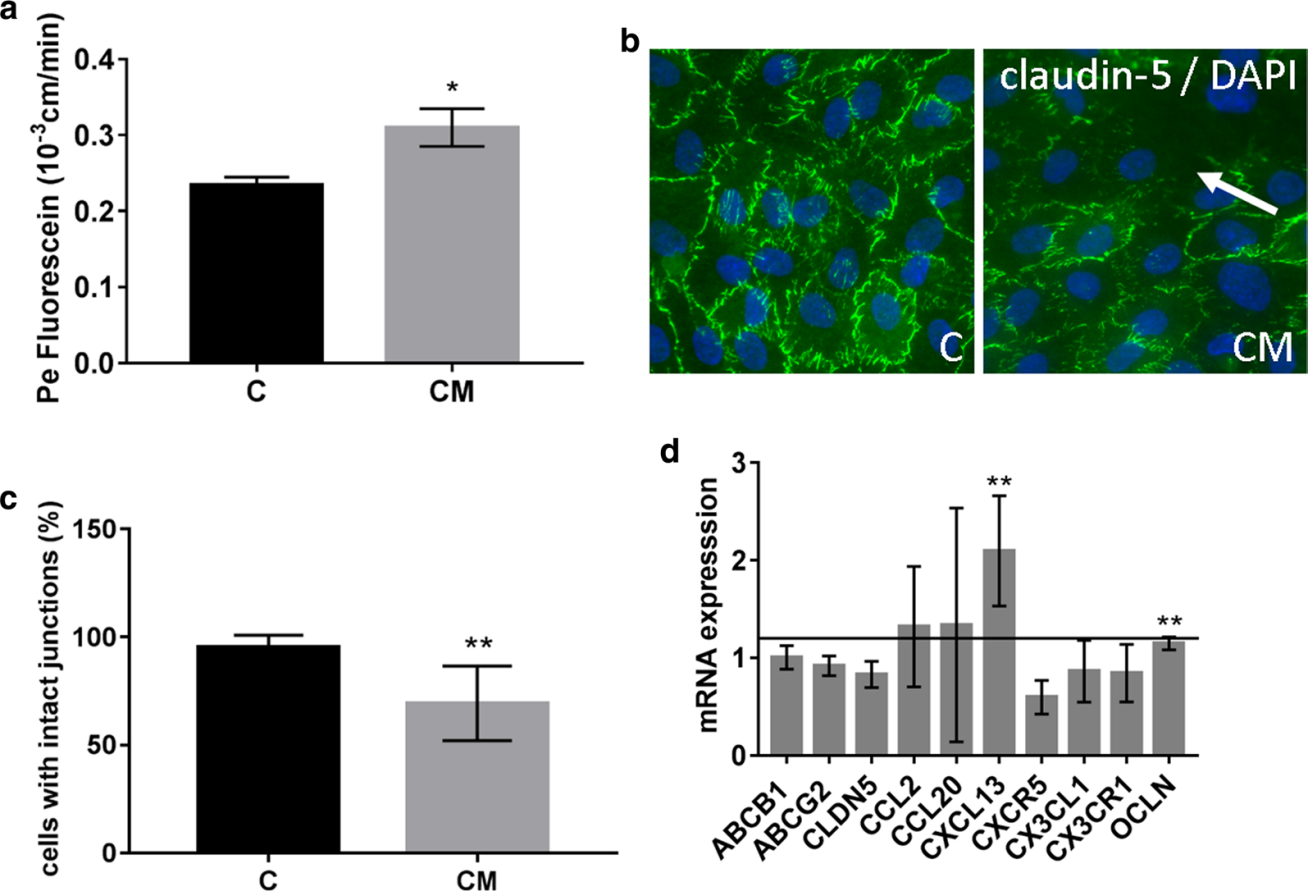
BLECSs treated with serum from patients with cerebral metastases show decreased barrier properties. BLECS were co-cultured with brain pericytes for 6 days in a transwell system. **a** BLECs were treated with the control sera (C) and sera from breast cancer patients with cerebral metastases (CM) for 24 h followed by measuring the paracellular permeability for fluorescein. Data are shown as mean ± SD of three independent experiments, *p < 0.05. **b** Immunostaining of claudin-5 in BLECs co-cultured with pericytes and treated either with serum from healthy donors (C) or breast cancer patients with cerebral metastases (CM). The arrow indicates loss of claudin-5 staining at the tight junctions of BLECs treated with serum from breast cancer patients with CM. Shown is one representative image of n = 6. Magnification ×400, green: claudin-5, blue: DAPI nuclear staining. **c** Quantification of cells with intact claudin-5 junctions presented in B. **d** Messenger RNA expression of chemokines and BBB-markers in BLECs treated with control serum and serum from breast cancer patients with cerebral metastases. The mRNA was quantified by qPCR. The target gene expression was normalized to endogenous control and shown as fold over control, which was arbitrarily set as 1 (control level marked in graph). Data are shown as mean ± SD of n = 4 (control sera) and n = 5 (CM). **p < 0.01 statistically significant versus control. C-X-C Motif Chemokine Receptor 5 (CXCR5), C-X3-C Motif Chemokine Receptor 1 (CX3CR1)

**Fig. 5:**
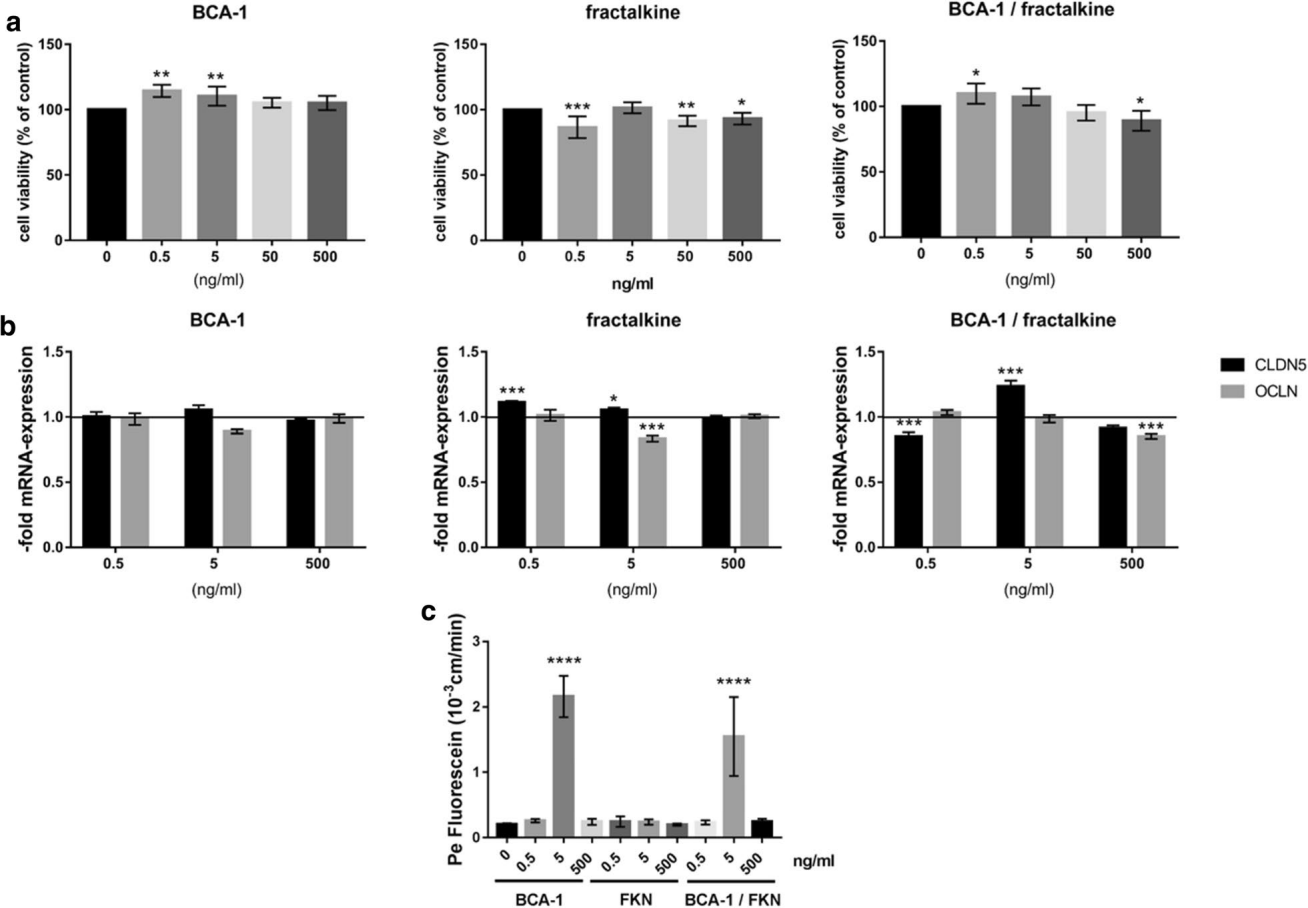
Effects of recombinant BCA-1 and fractalkine treatment on BLECs. **a** BLECs were treated with indicated concentration of BCA-1, fractalkine or a combination of both for 24 h. Cell viability was assessed using MTT. **b** Messenger RNA expression of claudin-5 (CLDN5) and occludin (OCLN) in BLECS treated with indicated concentrations of BCA-1, fractalkine or a combination of both for 24 h. The target gene expression was normalized to endogenous control and shown as fold over control, which was arbitrarily set as 1 (control level marked in graph). Data are shown as mean ± SD. **c** Paracellular permeability for fluorescein of BLECs treated with indicated concentration of BCA-1, fractalkine (FKN) or a combination of both for 24 h. Data are shown as mean ± SD (n = 3). *p < 0.05, **p < 0.01, ***p < 0.001, ****p < 0.0001

After identifying two selectively elevated chemokines in the sera of breast cancer patients with brain metastases, we asked if such sera could affect BBB integrity. To test this hypothesis, we treated BLECs for 24 h with 2% control sera or sera from breast cancer patients with brain metastases (Fig. 4). Serum-derived factors significantly increased the paracellular permeability of the endothelial monolayer (Fig. 4a). In addition, staining of serum-treated cells with anti-claudin-5 antibody revealed areas with reduced claudin-5 staining within the monolayer, suggesting a leaky barrier at that site (Fig. 4b, white arrow). Next, we analysed the mRNA expression of chemokines and BBB-markers after serum treatment (Fig. 4c). We detected a significant increase in occludin and BCA-1 mRNA levels in cells treated with sera of breast cancer patients with cerebral metastases compared to cells treated with the control serum. Other markers and chemokines such as ABCB1, ABCG2, claudin-5, CCL2, CCL20, CXCR5 (CXCL13 receptor), CX3CL1, CX3CR1 (CX3CL1 receptor) were not significantly altered (Fig. 4c). These results suggest that even low levels of patient serum (2%) can increase paracellular permeability and alter the expression and cellular localization of TJ proteins responsible for sealing of the BBB. We next examined the effects of recombinant BCA-1 and fractalkine on BLECs (Fig. 5). We tested the dose-dependent effects of both chemokines on cell viability using MTT assay (Fig. 5a). BCA-1 led to an increased cell proliferation in concentrations of 0.5 and 5 ng/ml. This effect was also observed in combination with 0.5 ng/ml fractalkine. Fractalkine alone showed inhibitory effects on cell viability at a concentration of 0.5, 50 and 500 ng/ml, similar to BCA-1/fractalkine in concentration of 500 ng/ml. We measured the CLDN5 and OCLN mRNA expression in cells treated with 0.5, 5 and 500 ng/ml BCA-1 or/and fractalkine. A significant increase in CLDN5 mRNA was observed in samples treated with 0.5 ng/ml fractalkine and 5 ng/ml BCA-1/fractalkine. The OCLN mRNA was reduced after treatment with 5 ng/ml fractalkine and 500 ng BCA-1/fractalkine (Fig. 5b). In addition, we measured the permeability to fluorescein in cells treated with 0.5, 5 and 500 ng/ml BCA-1 or/and fractalkine (Fig. 5c). Interestingly, after a treatment with 5 ng/ml BCA-1 alone and in combination with fractalkine, we found a strong increase in the paracellular permeability. Chemokines at the lowest concentration, which was similar to the chemokine level measured in patient sera, had no effect on the paracellular permeability.

## Discussion

The integrity of the BBB is a key feature responsible for protecting the brain from harmful substances, regulating entry and efflux of macromolecules and immune cells to and from the brain, and maintaining homeostasis of the CNS [32, 33]. Inflammation can affect the barrier properties of the BBB. However, clinical and in vitro data on inflammation and underlying pathomechanisms at the BBB in breast cancer patients with cerebral metastases are limited. Therefore, we designed the present study to analyse serum-derived chemokines in breast cancer patients with different cancer characteristics and to test the effects of patient sera on the BBB properties in vitro.

We utilized the CD34^+^ cells-derived in vitro BBB model first published by Cecchelli et al. in 2014 [12]. Similar to the first report on this model, we observed the induction of TJ proteins, multiple transporters and cellular receptors either at the protein or mRNA level when the cells were co-cultured together with pericytes. Pericytes have been chosen, because they induce BBB characteristics in CD34^+^ cells-derived hematopoietic stem cells [12] and they are known to play a role in the maturation and stabilization of the BBB [34, 35]. Induction of TJ-protein expression was accompanied by low paracellular permeability for fluorescein. The permeability values were similar to previously published values for this [12] and also for other in vitro BBB models [36]. However, fluorescein is a transport substrate for organic anion transporter 3 (OAT3) and multidrug resistance protein 2 (MRP2) [37]. These two transporters act in the mammalian BBB in the basolateral-to-apical direction, therefore they limit the apical-to-basolateral flux of fluorescein. This could lead to an underestimation of the paracellular fluorescein diffusion in our model system. However, the differences between the control and the treated cells should still be clearly visible. It can be assumed that a 6 day-co-culture with brain pericytes is sufficient to induce BBB properties in CD34^+^ cells-derived hematopoietic stem cells. Differentiated BLECs can be used to study molecular mechanisms underlying brain disorders, such as brain metastases.

In general, inflammatory mediators are mainly expressed by macrophages such as microglia, but also by astrocytes, oligodendrocytes and vascular endothelial cells [38]. Numerous mediators play a role in this out-balanced process, including anti-inflammatory cytokines, pro-inflammatory cytokines, chemokine-ligands and receptors. A correlation between cancer metastases and cytokine expression is discussed for various tumour entities [39, 40], including breast cancer [41, 42]. Therefore, we examined chemokines in the sera of breast cancer patients with and without cerebral metastases in comparison to healthy donors. Among the chemokines tested, CX3CL1 and CXCL13 were selectively and significantly increased in patients with cerebral metastases of breast cancer. The chemokines levels correlated with the tumour properties and the presence of other chemokines in patient sera. We identified a statistically significant correlation between RANTES and MCP-1 as well as BCA-1 and CCL20. RANTES and MCP-1 co-expression in breast cancer was associated with more advanced stages of the disease [43]. High CCL20 levels contribute to the formation of bone metastases in breast cancer [44]. CX3CL1 (also known as fractalkine or neurotactin) is a membrane-bound chemokine that can facilitate intercellular interactions, interacts with the TNFα-converting enzyme ADAM17 and is released in its shed form by apoptotic cells to recruit professional phagocytes to the site of cell death [45, 46]. Fractalkine serum concentrations were higher in patients with the ER/PR negative tumours, which is in line with the literature. Andre et al. showed in a study with 142 patients that a high CX3CL1 expression in the primary cancer correlates with brain metastases in a 13-year median-follow up [47]. Similarly, Tsang et al. postulated that CX3CL1 expression is associated with poor outcome in breast cancer patients [48]. High levels of CX3CL1 in cells can attract those cancer cells expressing its receptor CX3CR1 and trigger them to invade the tissue and form metastases as seen e.g. in breast cancer spinal metastases [49]. In our study, we showed that CX3CL1 is selectively elevated in the serum of breast cancer patients with cerebral metastases. CX3CL1 may therefore be involved in the formation of metastases in the brain, but further investigations are needed to fully elucidate the underlying mechanisms. Another elevated chemokine in the serum of breast cancer patients with brain metastases was CXCL13 (also known as B cell-attracting chemokine 1, BCA-1). Our results are consistent with other reports showing elevated BCA-1 serum concentrations in patients with metastatic disease [50]. A negative correlation between BCA-1 and the histological grading in patients with brain metastases indicates that high BCA-1 serum concentrations can lead to brain metastases from moderately differentiated tumours with low histological grading. However, our analysis is limited by a small number of samples. In breast cancer cell lines, CXCL13 induced changes of epithelial-to-mesenchymal transition marker expression. It upregulated vimentin, Snail, Slug, N-cadherin, MMP9 and RANKL and downregulated E-cadherin [51]. The endothelial-to-mesenchymal transition of brain endothelial cells has been described [52] and could also play a role in our in vitro model. We detected the mRNA of CXCR5 and CX3CR1 in BLECs, which are chemokine receptors for BCA-1 and fractalkine, respectively. The presence of mRNA expression of these receptors also indicates protein expression in BLECs. The receptors can bind BCA-1 and fractalkine present in sera or cell culture medium and transmit their signals. However, the cells treated with recombinant BCA-1 and fractalkine showed an increase in paracellular permeability only at high chemokine concentrations, with no effects in the concentration range observed in patient sera. This suggests that the BCA-1 and fractalkine require other synergistic factors in serum to affect the barrier. Induction of CXCL13 and CX3CL1 accompanied by compromised barrier integrity, has been reported in brain endothelial cells that overexpress claudin-1 and during ischemia/reperfusion injury in animal models [53]. It appears that CXCL13 and CX3CL1 are part of the endothelial inflammatory phenotype that plays a role in various cellular processes.

One of the limitations of our study may be the moderate number of serum samples taken from breast cancer patients, especially those with cerebral metastases. It will be interesting to increase the number of samples for chemokine analyses and to further evaluate serum effects on BBB properties. In vitro BBB models are valuable tools for studying cellular responses to factors such as patient sera. However, we need to consider that these are model systems that do not fully restore the microenvironment present in the human body.

## Conclusion

The CD34^+^ cell-derived human in vitro BBB model shows high barrier properties, accompanied by the expression of BBB and endothelial cell markers. It was used in the present study to analyse the mechanisms underlying the formation of cerebral metastases in breast cancer. Sera with elevated CX3CL1 and CXCL13 levels displayed barrier-compromising effects in vitro and therefore could contribute to the formation of brain metastases by breast cancer cells in vivo.

## Data Availability

The datasets used and/or analysed during the current study are available from the corresponding author on reasonable request.
